# The supergiant amphipod *Alicella gigantea* may inhabit over half of the world’s oceans

**DOI:** 10.1098/rsos.241635

**Published:** 2025-05-21

**Authors:** Paige J. Maroni, Yakufu Niyazi, Alan Jamieson

**Affiliations:** ^1^ School of Biological Sciences, The University of Western Australia, Perth, Western Australia, Australia; ^2^ Minderoo-UWA Deep-Sea Research Centre, The University of Western Australia, Perth, Western Australia, Australia

**Keywords:** Amphipoda, Alicellidae, deep sea, geographic range, abyssal, hadal

## Abstract

The deep-sea amphipod *Alicella gigantea* Chevreux, 1899, currently known as the world’s largest amphipod, inhabits depths of the lower abyssal and upper hadal zones. Historically, it has been sampled or observed *in situ* infrequently relative to other deep-sea amphipods, suggesting low population densities and providing a sense of rarity. Consequently, little is known about the demography, genetic variation and population dynamics of *A. gigantea*, with only seven studies having published DNA sequence data. As more records emerge from across the vastness of the deep sea, and from depths beyond most conventional sampling, there is an ever-growing body of evidence to show that *A. gigantea* should be considered far from rare. In this study, we compile 195 records of *A. gigantea* from 75 locations worldwide and use two mitochondrial genes and one nuclear gene (*16S*, *COI* and *28S*) to explore their distribution patterns across all oceans and discuss the species history throughout geological time. Our results show that this species may occupy around 59% of the world’s oceans, indicating that the infrequently collected supergiant is not ‘rare’ but instead represents a widely distributed deep-sea amphipod with an exceptional global range.

## 1. Introduction

Amphipods (Arthropoda: Crustacea: Amphipoda) inhabit all aquatic environments worldwide and are among the most specious and ecologically diverse orders of crustaceans encompassing over 10 000 extant species [1]. Their ubiquity extends to the deep sea, particularly the abyssal (3000−6000 m) and hadal environments (6000 to ~11 000 m); however, the number of species generally decreases with depth [2]. At hadal depths, it was determined that 83% of the more than 100 known amphipod species were recorded from one to two hadal features [3]. The most conspicuous families, in terms of diversity and geography, are Alicellidae, Pardaliscidae, Hirondellidae and Scopelocheiridae, although these families contain many global species [4–6] as well as many seemingly ‘rare’ species [3]. Within these infrequently collected species, there are examples where the body size is sufficiently prominent to question why such species are not collected or observed more often. A prime example is *Alicella gigantea* Chevreux, 1899 [7], commonly known as the ‘supergiant amphipod’ and recognized as the world’s largest amphipod species. This species has attracted widespread attention due to its significant gigantism with a maximum body length of 340 mm [8–10].

*Alicella gigantea* was first filmed *in situ* at 5304 m in the abyssal North Pacific [11], although at the time it was not known what species it was. The finding of an amphipod estimated to be over 28 cm long was sufficiently interesting to warrant a publication in the journal *Science*. Eventually, a few captured specimens were identified as *A. gigantea* [8]. Then, for over two decades, no records of *A. gigantea* were made until it was filmed *in situ* and recovered from hadal depths in the Kermadec Trench in the South Pacific (6265−7000 m) [9]. During the recent renaissance in exploration and sampling at hadal depths an ever-increasing number of observations and samples are emerging from disparate locations spanning several oceans [3]. The supergiant amphipod is now known to be present at eight hadal features across the Pacific, Indian and Atlantic oceans.

Regardless of this widespread distribution, these animals have historically been collected in small numbers, perhaps signifying low population densities and providing a sense of rarity [9,10]. As a result, little is known about the demography, genetic variation and population dynamics of *A. gigantea* with only seven studies having published DNA sequence data [9,10,12–16]. As more records emerge across the vastness of the deep sea, and from depths that are beyond conventional sampling depths, there is an ever-growing body of evidence to show that *A. gigantea* should be considered far from rare. While their population density may be low relative to other deep-sea amphipods, they appear to inhabit an extraordinarily large geographical range, which questions whether they are represented by one or multiple species.

In this study, we compile all records of *A. gigantea* and sequence newly collected specimens using two mitochondrial genes and one nuclear gene (*16S*, *COI* and *28S*) to explore the phylogeography of *A. gigantea* across several oceans, further examine the distribution of this species and discuss the evolutionary history throughout geological time. These molecular markers were investigated to compare all newly collected specimens to published datasets to formulate a global species hypothesis for future testing. We also incorporated the mitochondrial genome of *A. gigantea,* utilized habitat mapping techniques, a species delimitation tool and investigated haplotype and phylogenetic data to better understand this species’s contemporary distribution.

## 2. Material and methods

### 2.1. Biogeographical data

**Figure 1.**
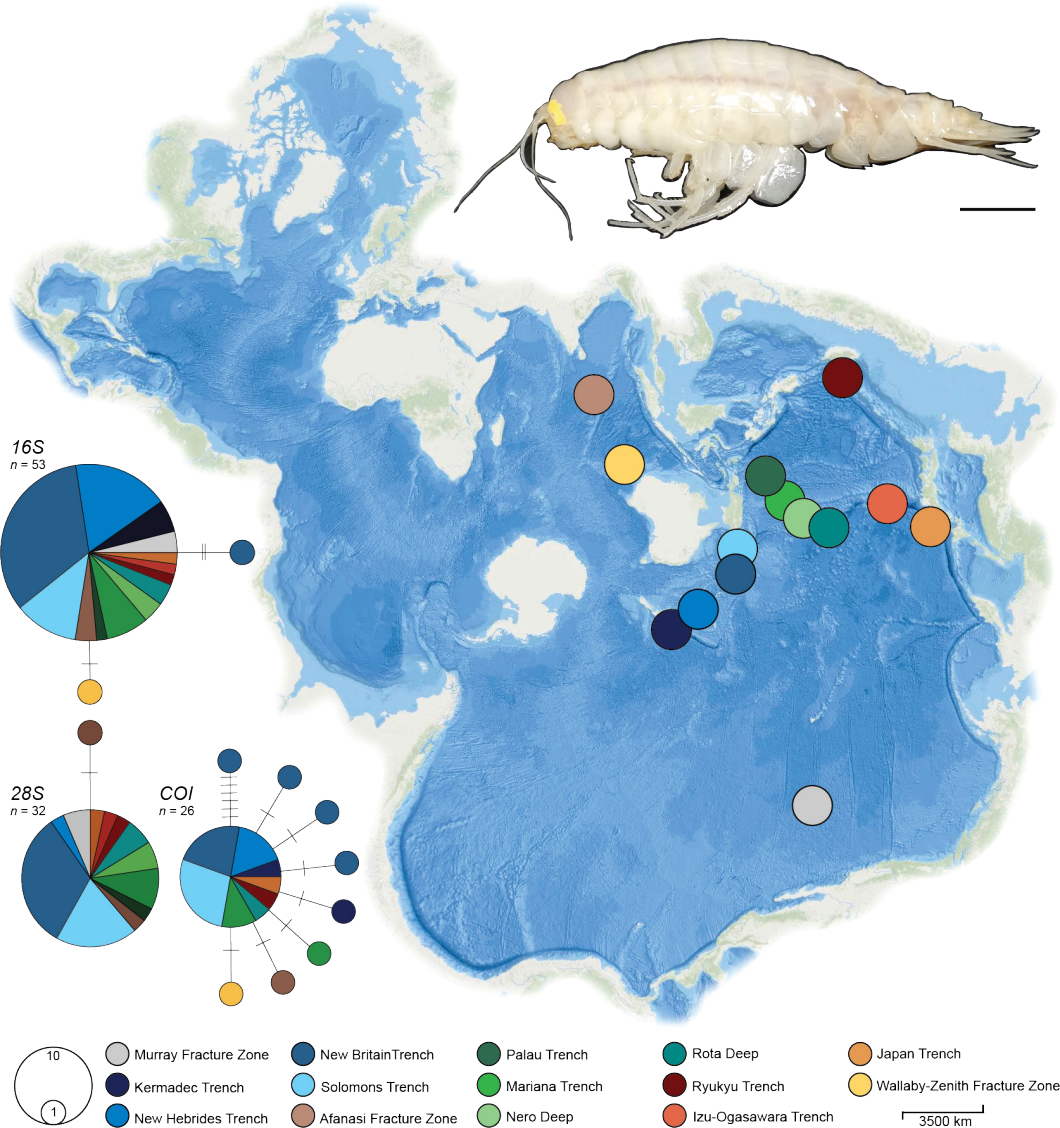
*Top right*: Fresh caught specimen of the supergiant amphipod *A. gigantea*, from 6746 m in the Murray Fracture Zone, North Pacific Ocean. Scale: 2 cm. *Middle*: Spilhaus world projection showing the sample sites of all sequenced specimens used in this study. Each site is denoted by a circle and colour-coded by deep-sea feature. *Bottom left*: Three TCS haplotype networks depicting the sequenced material. The area of each circle is proportional to the frequency of the haplotype and the colours represent the location from which corresponding samples were collected.

Geographical data from *in situ* observations and collections (included instances of both) were compiled from 18 published articles [7–12,15–26]. Combined, these studies span 15 different seafloor features across the Pacific, Atlantic and Indian Oceans. To date, there are no records from the Arctic or Antarctic oceans. New, unpublished records from the Five Deeps and Ring of Fire Expeditions on the DSSV *Pressure Drop* (Caladan Oceanic LLC) were pooled from the Mariana, Palau, Santa Cruz, San Cristóbal, Tonga, Ryukyu, Izu-Ogasawara and Japan trenches and more recent samples and records collected by the same vessel (renamed RV *Dagon*; Inkfish) from the Murray Fracture Zone, NE Pacific Ocean during the 2023 Trans-Pacific Expedition [27] were also included (figure 1). Additional samples were also obtained from the RV *Sonne* cruise (SO259) from the Zenith Plateau and Afanasi Nikitin Seamount in the Indian Ocean [15] and from the 2012 RV *Mermaid Sapphire* cruise from the New Britain Trench in the SW Pacific Ocean (electronic supplementary material, table S1). It is important to note here that most of the sampling sites are concentrated in the Pacific Ocean, with limited representation from the Atlantic and Indian Oceans.

### 2.2. Sample preservation

The newly sequenced specimens were collected using autonomous lander systems that featured invertebrate and small vertebrate traps baited with whole mackerel (Scombridae). These lander systems were also equipped with high-definition (HD) video cameras (IP Multi SeaCam 3105; Deep Sea Power and Light, San Diego, CA, USA) that continuously recorded, as well as pressure and temperature sensors (SBE 49 FastCAT, SeaBird Electronics, Bellevue, WA, USA), which recorded data at 10 s intervals. Tissue sub-samples were extracted from freshly caught specimens that were then preserved in 96–100% ethanol. Comprehensive data, including deployment specifics, collection details and sequence accession information for all specimens, can be found in electronic supplementary material, table S2.

### 2.3. DNA extraction, PCR amplification and DNA sequencing

Total genomic DNA was extracted from 31 ethanol-fixed samples using a DNeasy Blood and Tissue Kit (Qiagen) according to the manufacturer’s instructions for tissue samples. Two mitochondrial barcoding regions, 16S rRNA (*16S*) and cytochrome oxidase I (*COI*) and one nuclear locus, 28S rDNA (*28S*) were amplified using published primer sets. For each PCR reaction, 7.5 μl of molecular grade (deionized) water, 2 μl forward primer, 2 μl reverse primer and 12.5 μl of AmpliTaqGold 360 Master Mix (Thermo Fisher Scientific) were included with 2 ng of DNA template added for *16S,* and 20 ng of template used for and *28S* and *COI*. The following PCR conditions were used for amplification of the *16S* fragment: 7 min at 95°C, 12 cycles of 30 s at 95°C, 30 s at 62°C (decreasing one degree after each cycle), 1 min at 72°C, 29 cycles of 30 s at 95°C, 15 s at 51°C, 1 min at 72°C with a final extension time of 10 min at 72°C on a Veriti Thermal Cycler (Thermo Fisher Scientific). Primers for *16S*: AMPH1 (5′−GAC GAC AAG ACC CTA AAA GC−3′) [28] and ‘Drosophila-type’ 16SBr (5′−CGG GTT TGA ACT CAG ATC ATG−3′) [29]. PCR conditions for amplification of *28S*: 7 min at 95°C, 12 cycles of 30 s at 95°C, 30 s at 62°C (decreasing one degree after each cycle), 1 min at 72°C, 35 cycles of 30 s at 95°C, 15 s at 52°C, 1 min at 72°C with a final extension time of 10 min at 72°C. Primers for *28S*: 28Sftw (5′−AGG CGG AAT GTT GCG T−3′) and 28Srtw (5′−CTG AGC GGT TTC ACG GTC−3′) [30]. For *COI,* the thermocycling conditions were set as: 7 min at 95°C, 12 cycles of 30 s at 95°C, 30 s at 56°C (decreasing one degree after each cycle), 1 min at 72°C, 35 cycles of 30 s at 95°C, 30 s at 45°C, 1 min at 72°C with a final extension time of 10 min at 72°C. Primers for *COI*: LCO1490 (5′−GGT CAA CAA ATC ATA AAG ATA TTG G−3′), HCO12198 (5′−TAA ACT TCA GGG TGA CCA AAA AAT CA−3′) [31]. Amplicons were initially examined using 96-well E-gels (Invitrogen) and subsequently purified with AMPure XP paramagnetic beads (Beckman and Coulter). Following this, the samples were prepared for sequencing reactions employing the Thermo Fisher Scientific Applied Biosystems BigDye Cycle Sequencing Kit. Sequencing reactions were cleaned using the CleanSEQ Dye-Terminator Removal Protocol provided (Beckman Coulter) and were subsequently sequenced using a 3730xl capillary sequencer (Thermo Fisher Scientific Applied Biosciences), situated at the Australian Genome Research Facility (AGRF) in Perth, Western Australia. All sequences were assembled and edited in Geneious Prime 2023.0.1 [32].

### 2.4. Phylogenetic reconstruction

To infer genetic relationships, a dataset for each molecular marker and a concatenated dataset of all loci were generated, and all trees were rooted with *Tectovalopsis* species [26,33]. All ingroup sequences generated here were mapped to the mitochondrial genome of *A. gigantea* for species identification [10]. Published ingroup sequences [9,12–15] were also downloaded from GenBank and incorporated for phylogenetic analysis. All datasets were aligned with the MAFFT plugin for Geneious Prime (v. 1.5.0; https://www.geneious.com/plugins/mafft-plugin) [34] using default settings. All alignments were used to create maximum likelihood (ML) phylogenies using IQ-TREE2 (v. 2.2.0; http://www.iqtree.org) [35], and within these analyses, nodal support was assessed with 10 000 ultrafast bootstrap replicates [36]. For the ML analyses, the -m TEST [37] option in IQ-TREE2 identified HKY+F+I for the *16S* dataset, JC for the *28S* alignment and HKY+F+G4 for the *COI* alignment as the best-fit models chosen according to Bayesian Information Criterion (BIC). These best-fit models were then used as input to inform a partitioned complex ML model for the concatenated alignment within IQ-TREE2. Each alignment was also subject to a Bayesian analysis, carried out with the Geneious Prime MrBayes plugin (v. 3.2) [38,39]. A total of four independent Markov chain Monte Carlo runs were performed, and each run was composed of four heated chains (chain temperature = 0.2). Each analysis was run for 40 000 000 generations, with a burn-in length of 4 000 000 and a sub-sampling frequency of 10 000 under a HKY85 model of evolution. Convergence of the runs was determined by examining the average standard deviation of split frequencies using Tracer [40] within the Geneious Prime, MrBayes plugin.

To calculate intra- and interspecific genetic distances, all outgroup specimens were removed for model accuracy and the pairwise distances (uncorrected *p*-distances) or the best-fit corrected genetic distances, chosen according to BIC, were calculated. Distances were calculated for each dataset using the *dist.dna* function from the R package Ape (v. 5.6; https://cran.r-project.org/web/packages/ape/index.html) [41] with either ‘raw’ or the best-fit model specified as the evolutionary model. For *16S* and *COI,* the F81 [42] corrected genetic distances were calculated and for *28S* the JC69 [43] distances were calculated. The base frequencies were specified for the corrected genetic distance calculations (*16S: A* = 0.276, *C* = 0.118, *G* = 0.261, *T* = 0.344; *COI: A* = 0.269, *C* = 0.213, *G* = 0.173, *T* = 0.345; *28S: A* = 0.288, *C* = 0.225, *G* = 0.281, *T* = 0.206). To address the lack of morphological comparisons in this study, we also tested our species hypothesis using the ascending hierarchical clustering program, Assemble Species by Automatic Partitioning (ASAP) [44]. All corrected genetic distances, per loci, were imported into ASAP (substitution mode = JC69 [43]) for comparison.

### 2.5. Haplotype structure, diversity estimates and demography

To visually inspect the geographic structure of haplotypes, three single-loci TCS haplotype networks were constructed in PopART [45] with a 95% probability threshold. For each network, locality data was overlaid, and in total, 14 geographical regions were defined *a priori* (figure 1) to visualize genetic patterns across sampling space. Levels of polymorphism in the data were represented by haplotype number (*H*), haplotypic (*h*) and nucleotide diversity (π) indices and number of segregating sites (*ss*). All values, per loci, were estimated using the R packages ‘pegas’ [46] and ‘ape’ [41] (table 1). To infer historical population changes and/or deviations from neutrality, we analysed Tajima’s *D* [47] also using ‘ape’ [41].

**Table 1.** Genetic diversity statistics for A. gigantea. **Statistical significance at *p ≤ *0.001.

	*16* ***S***	*COI*	*28* ***S***
*n*	53	26	31
sample sites	14	10	11
haplotype count (*H*)	3	9	3
haplotype diversity (*h*)	0.44267	0.58154	0.36129
nucleotide diversity (*π*)	0.0006	0.00184	0.00024
segregating sites (*ss*)	5	14	1
corrected genetic distance	0.000–0.016%	0.000–0.0.013%	0.000–0.327%
Tajima’s *D*	−1.60894	−2.43648**	−1.54226

### 2.6. Habitat mapping

Given that depth has a strong impact on environmental conditions such as pressure, temperature and oxygen availability, and that little is known about *A. gigantea’s* ecological and biological associations, depth was considered the primary ecological factor influencing its distribution. Therefore, depth was used as a proxy for habitat in our study. Depth data was extracted from 75 sites across all *A. gigantea* records, ranging from 3890 to 8931 m, which defined the potentially suitable habitat for *A. gigantea*. The total areal coverage of this depth range within the world’s six main water bodies (five oceans and the Mediterranean Sea) was calculated using the General Bathymetric Chart of the Oceans [48] gridded bathymetry data at approximately 450 m resolution. The boundary of the world oceans was based on the Global Oceans and Seas of Flanders Marine Data Centre (https://marineregions.org). The bathymetry was first reclassified by the depth ranges and then converted to polygons to calculate the area using world Mollweide projection in ArcGIS Pro (v. 3.3; https://www.esri.com/en-us/arcgis/products/arcgis-pro/overview). Global palaeo-topography and palaeo-bathymetry were also reconstructed to visualize the expanse of the deep sea for three intervals across the past 100 million years. The palaeo-digital elevation model [49] datasets from 100 Ma, 50 Ma and present-day were downloaded as text datasets (accessed from https://www.earthbyte.org/paleodem-resource-scotese-and-wright-2018) and were reconstructed in R using the package *ggplot2* [50].

## 3. Results

### 3.1. Divergence

The phylogenetic reconstruction, based on mitochondrial (*16S* rRNA and *COI*) and nuclear (*28S* rDNA) loci, identified clear genetic relationships among the *A. gigantea* specimens collected from all locations. The ML and Bayesian analyses produced consistent topologies, with strong nodal support (electronic supplementary material, figure S1). As our study was limited by a lack of morphological comparisons, intraspecific genetic distances and the species delimitation tool ASAP were investigated to assess this diversity among loci and among samples. The *COI* distances varied the most within the dataset, however, all genetic distances suggested a high degree of conservation within *A. gigantea*, with limited divergence across the world’s oceans (table 1; electronic supplementary material, figure S2). The ASAP species hypothesis partition, ranked by the best ASAP score, delimited two species for the *16S* (ASAP score = 1.00, *p* = 0.0621) and *COI* (ASAP score = 1.00, *p* = 0.0693) datasets, and one species for the *28S* dataset (ASAP score = 1.50, *p* = 0.814). In the *16S* and *COI* datasets, the single sample partitioned as its own distinct 'species' was CNBT3, which, according to [12], is a population of *A. gigantea* rather than a separate species.

### 3.2. Distribution

Haplotype networks constructed for each locus indicated low levels of genetic polymorphism, with large, shared haplotypes across different geographic regions (figure 1), supporting the hypothesis that this species’ distribution is vast and continuous. Specifically, the *16S* haplotype network consisted of one large, shared haplotype and two private (only found at a single location) haplotypes, the *COI* network consisted of a shared haplotype and eight private haplotypes and the *28S* network was made up of one large, shared haplotype and a single, private haplotype. The haplotype and nucleotide diversity indices (*H*, *h*, *π*) and the number of segregating sites (*ss*) reflected low-to-moderate genetic variation, suggesting limited regional differentiation (table 1). A significant negative Tajima’s *D* value was detected for *COI*, suggesting that the populations may be experiencing growth, purifying selection (i.e. deleterious mutations are being removed) or a selective sweep/positive selection, resulting in more low-frequency variants than expected under neutral evolution. Negative, however, non-significant Tajima’s *D* values were also detected for *16S* and *28S* (table 1). Overall, *COI* was the most variable loci investigated in terms of haplotype diversity, nucleotide diversity, segregating sites and Tajima’s *D,* whereas *28S* was the least variable.

**Figure 2.**
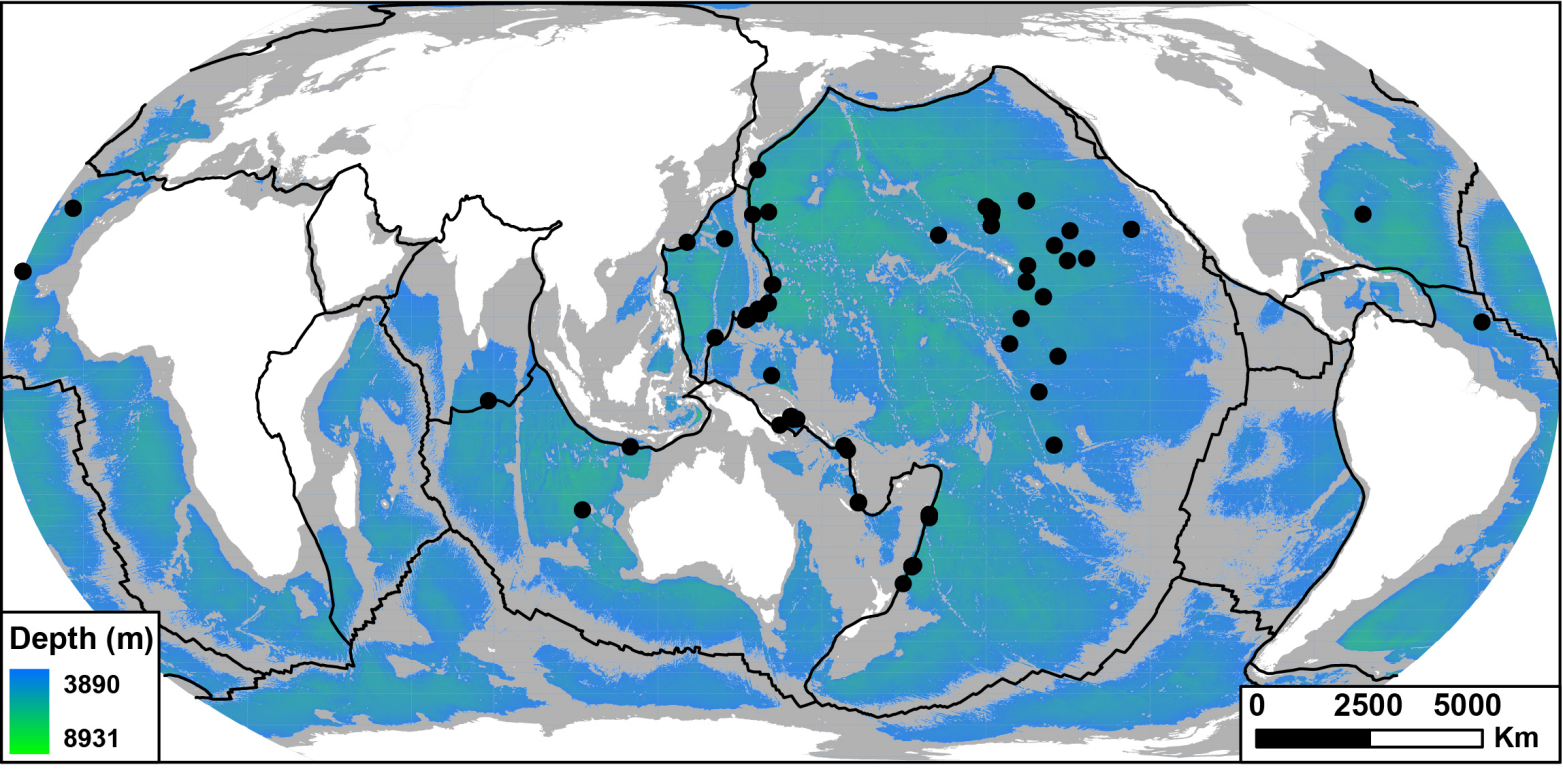
*Alicella gigantea* sampling sites (black dots) and GEBCO_2023 bathymetry (colour) of the world ocean for water depths between 3890 and 8931 m, representing the geographical distribution of *A. gigantea*. Black lines represent first-order plate boundaries (adopted from [51])*.*

A total of 75 sites were analysed to extract depth data for *A. gigantea*, revealing a depth range between 3890 and 8931 m. This range was utilized to define the potential habitat of *A. gigantea* across the world’s oceans. Despite the absence of records from the Arctic, Antarctic and Mediterranean, our global depth-based habitat suitability projection suggests that *A. gigantea* could inhabit approximately 59% of the world’s oceans and all six major ocean bodies (figure 2 and table 2).

**Table 2.** Global area calculations corresponding to the projected distribution of *A. gigantea*. The first column indicates the total area by ocean that is deeper than 3890 m and shallower than 8931 m. The second column represents the total km^2^ that ocean makes us globally. The third column represents what percentage of the total ocean area is within *A. gigantea*’s depth range.

	area (km^2^) 3890−8931 m	total ocean area (km^2^)	area (%) within *A. gigantea* depth range
Pacific Ocean	104 575 622	140 274 790.80	74.55%
Atlantic Ocean	47 677 056.41	82 348 325.50	57.89%
Indian Ocean	39 622 525.03	70 834 789.42	55.93%
Southern Ocean	8 443 960.06	29 594 289.41	28.53%
Arctic Ocean	564 328.79	11 880 270.28	4.75%
Mediterranean Sea	28 810.49	2 988 248.07	0.96%
total	200 912 302.80	337 920 713.50	59.45%

Specifically, the Pacific Ocean was identified as the largest potential habitat for *A. gigantea*, encompassing approximately 104.6 million km², which constitutes 75% of its total surface area within the species's depth range (table 2). This was followed by the Atlantic Ocean, which provides around 47.7 million km² of suitable habitat, accounting for 58% of its oceanic area. Similarly, the Indian Ocean offers about 39.6 million km² of suitable habitat, covering 56% of its total area. It is important to note, however, that of the 75 sites included in this analysis, 67 are from the Pacific Ocean, four are from the Indian Ocean and four are from the Atlantic Ocean, which may influence these results. Also, samples collected from the Pacific Ocean represent the shallowest and deepest samples of *A. gigantea* included within this study. In contrast, the Southern Ocean contains up to 8.4 million km² of potential habitat, representing 29% of its surface area, while the Arctic Ocean provides only 0.6 million km², or 5% of its area. The Mediterranean Sea was found to have the smallest extent of suitable habitat, covering approximately 0.03 million km², which is equivalent to just 1% of its enclosed ocean surface.

## 4. Discussion

The significant finding of this work is that *A. gigantea* is a single, globally distributed deep-sea amphipod species. Here, we show that the ‘supergiant amphipod’, is now known from 75 sites, spanning the Pacific, Atlantic and Indian Oceans, with a depth range of 3890–8931 m. Sequenced specimens were collected from nine trenches and three fractures zones from the Pacific and Indian oceans, thus confirming that the contemporary distribution of this species is vast. To emphasize this, our habitat suitability model suggested that this species, may in fact inhabit up to 59% of the world’s oceans, with the Pacific Ocean representing the largest potential habitat. Haplotype networks showed low genetic polymorphism and minimal geographic differentiation, supporting this wide and continuous distribution.

### 4.1. Distribution and diversification through time

**Figure 3.**
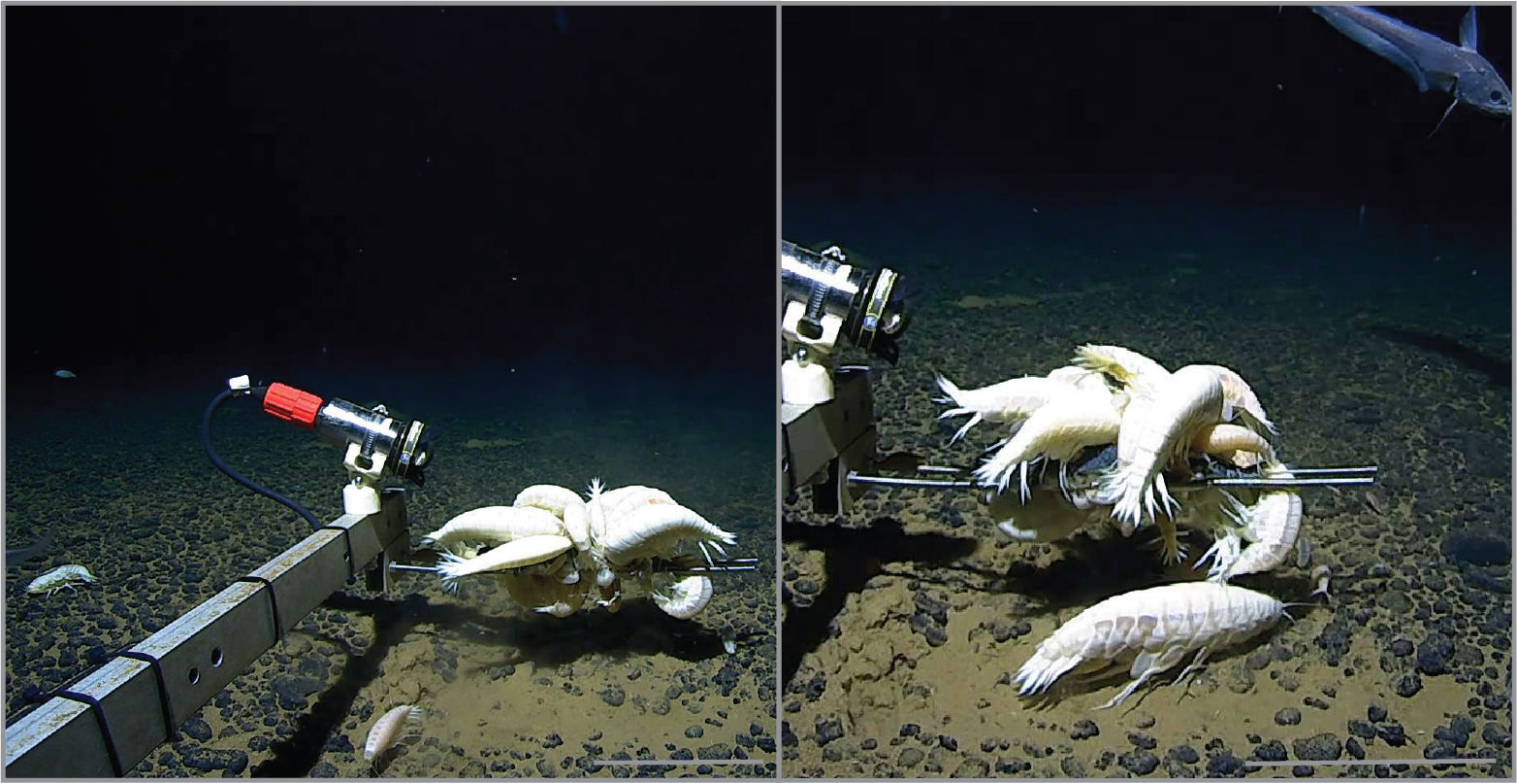
Aggregations of *A. gigantea* to bait at 6500–6700 m from the Murray Fracture Zone, North Pacific. Scale: 20 cm.

The contemporary distribution of *A. gigantea* and its predicted suitable habitat is predominantly segmented by tectonic boundaries and seafloor spreading centres. This punctuated, yet expansive range represents one of just three deep-sea amphipods species, considered to have a global distribution. *Hirondellea dubia* Dahl, 1959 [52] has been documented from eight deep-sea features [53] and *Bathycallisoma schellenbergi* Birstein & Vinogradova, 1958 [54] has been sampled from 16 features [5]. For these widespread deep-sea amphipod species, it was originally suggested that the observed genetic homogeneity among disjunct sites may be due to insufficient sampling across the adjoining abyssal plains at depths greater than 5000 m [3]. This study capitalized on a unique opportunity to access samples and video data collected during a large-scale abyssal plain project, specifically the Inkfish Trans-Pacific expedition [27]. With a dataset comprising 58 observations from 21 sites, we were able to intensively investigate the distribution of *A. gigantea*, demonstrating that multiple individuals—sometimes in high densities (figure 3)—inhabit the adjoining NE Pacific abyssal plains.

The analysis of depth data from 75 sites and the single, shared haplotypes which persist over ~8000 km (Kermadec Trench to Japan Trench) revealed that *A. gigantea* occupies a broad depth range (>5000 m vertical range), highlighting the species’ capacity to inhabit deep-sea environments across diverse oceanic regions. The habitat suitability projection (figure 2) suggested that this species could inhabit over half of the world’s oceans, covering all six major ocean bodies. It is important to consider the variation in sample depth ranges, particularly in the Pacific Ocean, where both the shallowest and deepest samples were collected. Although the uneven distribution of depth data may influence the habitat projections for regions such as the Southern Ocean, where no physical samples have been collected, the smaller proportion of available habitat in these regions may partially offset potential sampling bias.

While it is challenging to assess this species' contemporary connectivity pathways and population structure with the available dataset, we can still postulate how its vast distribution has been facilitated over time. The environmental shift from the Cretaceous Greenhouse to the Cenozoic Icehouse, punctuated by the Cenomanian–Turonian oceanic anoxic event (100–80 Ma) and the Palaeocene–Eocene Thermal Maximum (50 Ma) played a critical role in the colonization of the deep sea (below 1000 m) [55–58]. These climatic events coincided with the separation of Greenland from Europe and North America (130–80 Ma), and the break-up of Eastern Gondwana (80–60 Ma). These plate reconfigurations opened the oceanic gateways in the NE Atlantic and the Southern Ocean (i.e. the Drake Passage and the Tasman Gateway), enabling the formation of deep-water masses like the North Atlantic Deep Water and the Antarctic Circumpolar Current [59–66]. The latter allowed for the thermal isolation of Antarctica and triggered the onset of Antarctic glaciation around 45.5 Ma (intensifying ~34 Ma) [62]. Improved ocean circulation and ventilation during this time, allowed deep-sea species to expand their habitat and promoted multiple adaptive radiations [67], including the diversification of the Lysianassoid clade, which includes *A. gigantea*. These relatively recent radiations support the prevailing theory that most deep-sea species emerged during the Cenozoic (66 Ma–present), as the low oxygen conditions of the Mesozoic era—due to the warmer surface waters and higher CO_2_ level (especially during the Cretaceous Greenhouse period)—constrained diversification [57,68–70], supporting the global amphipod diversification hypothesis [68].

### 4.2. Larger, genomic datasets are required

Genetic diversity serves as a cornerstone for species’ survival and resilience in dynamic environments [71,72]. Within populations, genetic variation enables adaptation to varying ecological conditions and the ability to respond to environmental disturbances [73,74]. Moreover, the interconnectedness of populations through gene flow facilitates the exchange of genetic material, shaping the evolutionary trajectories of species [75,76]. Therefore, unravelling the genetic architecture and connectivity of these populations will provide valuable insights into the species’ adaptation and evolutionary potential in the face of changing environmental conditions.

To date, hadal-focused genetic analyses have predominantly utilized DNA barcoding methods to investigate fundamental taxonomy, biodiversity [9,14,15,74,77,78] and identify cryptic species [79–81]. Very few studies have directly examined patterns of population connectivity among hadal features [4,5,80,82]. One study on the populations of abyssal and hadal *Paralicella spp.* Chevreux, 1908 [83] utilized genome-wide markers and found sufficient gene flow among four Pacific Ocean trench populations, leading to the conclusion that trenches do not significantly restrict dispersal [4]. Conversely, the phylogeographic patterns of the amphipod *Bathycallisoma schellenbergi* Birstein & Vinogradova, 1958 [54] were investigated across 12 hadal features across four oceans using genome-wide single-nucleotide polymorphism (SNPs) markers and two mitochondrial regions [5]. This work found that despite its cosmopolitan distribution, the populations of *B. schellenbergi* showed strong restriction to individual features, with limited gene flow observed between topographically connected features. The authors noted that *B. schellenbergi* has not yet been found outside of trenches or fracture zones. This led them to conclude that the shallower abyssal regions separating the sampled hadal features have historically served as significant barriers to dispersal, resulting in genetic isolation [5]. Given the divergent findings from these two studies, it is imperative to continue to investigate population-level structure and connectivity across multiple species within the deep sea so we may better comprehend the role of these habitats in shaping deep-sea biodiversity and evolution. Similarly, collating large, disparate datasets on species occurrences, as exemplified in our study, provides a foundational framework for developing and testing hypotheses regarding patterns of cosmopolitism, endemism, rarity and their implications for the structure and connectivity of deep-sea ecosystems.

Population-level analyses, such as SNP-based research, greatly benefit from the availability of extensive genomic and transcriptomic data. Having more complete genomes and transcriptomes allows for a deeper investigation into the genetic variation within species, enabling researchers to identify key patterns of divergence, gene flow and adaptation across populations. These data are crucial for understanding evolutionary processes, as it provides insights into how selective pressures shape genetic diversity and the mechanisms underlying speciation. Moreover, broader genomic resources enhance the ability to detect signatures of natural selection and uncover the genetic basis of adaptations to specific environmental conditions, offering a more detailed understanding of species evolution and resilience. The number of available genomic resources for hadal amphipods remains quite limited, with only three mitogenomes [3,10,84,85] and three transcriptomes [16,86] published to date, thus it remains clear that across the field of deep-sea research, there is an extensive knowledge gap that requires attention. Expanding genomic resources for hadal amphipods faces significant challenges, including their exceptionally large genome sizes, estimated between 4 and 34 Gb [13]. Additionally, the drastic differences in pressure and temperature between the deep-sea environment and the surface cause rapid DNA degradation, leading to the swift deterioration of collected organisms. However, with rapid advancements in next-generation sequencing technologies and an increase in deep-sea exploration, research into the genomics, transcriptomics and evolution of hadal amphipods is expected to expand dramatically in the coming decades. These developments will help reveal further insights into hadal biodiversity, pressure adaptations and the evolutionary history of life in the hadal zone.

## 5. Conclusions

This study presents the largest and most extensive dataset ever compiled on the world’s largest species of amphipod, providing unparalleled insight into the vast geographic distribution of this cosmopolitan deep-sea species across the Pacific, Atlantic and Indian Oceans at depths ranging from 3890 to 8931 m. Genetic analyses revealed remarkably low divergence in *A. gigantea* populations worldwide, suggesting a high degree of conservation. Haplotype networks showed limited genetic polymorphism, with large, widely shared haplotypes spanning different regions, further reinforcing the idea of a vast and continuous distribution. Strikingly, a global depth-based habitat suitability model predicted that *A. gigantea* may inhabit roughly 59% of the world’s oceans. This finding confirms that the supergiant amphipod is far from ‘rare’ but instead represents a single, globally distributed species with an extraordinary and expansive range across the deep sea.

## Data Availability

DNA sequences, GenBank accession codes: COI: PQ345402-PQ345405, PQ345428-345433, 16S: PQ349560-PQ349590, 28S: PQ349529-PQ349559. Data available from Dryad [87]. Supplementary material is available online [88].
